# Toward a mechanistic understanding of electrocatalytic nanocarbon

**DOI:** 10.1038/s41467-021-23486-1

**Published:** 2021-06-02

**Authors:** Erik J. Askins, Marija R. Zoric, Matthew Li, Zhengtang Luo, Khalil Amine, Ksenija D. Glusac

**Affiliations:** 1 Department of Chemistry, University of Illinois at Chicago, Chicago, IL USA; 2 Chemical Sciences and Engineering Division, Argonne National Laboratory, Lemont, IL USA; 3 Chemical Engineering Department, University of Waterloo, Waterloo, ON Canada; 4 Department of Chemical and Biological Engineering, The Hong Kong University of Science and Technology, Clear Water Bay, Hong Kong, Hong Kong; 5 Department of Material Science and Engineering, Stanford University, Stanford, CA USA; 6 Institute for Research and Medical Consultants (IRMC), Imam Abdulrahman Bin Faisal University (IAU), Al Safa, Dammam, Saudi Arabia

**Keywords:** Electrocatalysis, Electrocatalysis, Electronic materials, Nanoscale materials

## Abstract

Electrocatalytic nanocarbon (EN) is a class of material receiving intense interest as a potential replacement for expensive, metal-based electrocatalysts for energy conversion and chemical production applications. The further development of EN will require an intricate knowledge of its catalytic behaviors, however, the true nature of their electrocatalytic activity remains elusive. This review highlights work that contributed valuable knowledge in the elucidation of EN catalytic mechanisms. Experimental evidence from spectroscopic studies and well-defined molecular models, along with the survey of computational studies, is summarized to document our current mechanistic understanding of EN-catalyzed oxygen, carbon dioxide and nitrogen electrochemistry. We hope this review will inspire future development of synthetic methods and in situ spectroscopic tools to make and study well-defined EN structures.

## Abstract (web summary)

Electrocatalytic nanocarbon (EN) is a class of materials receiving intense interest as next generation electrocatalysts. Although impressive platforms, work is still required to develop our mechanistic understanding of them to that of molecular electrocatalysts.

## Introduction

**Fig. 1:**
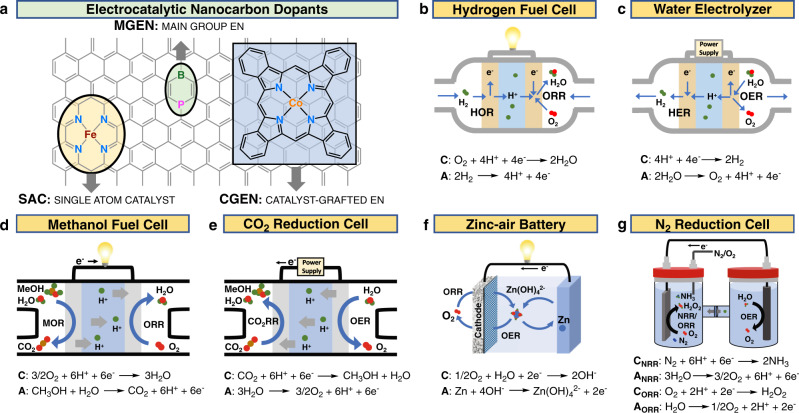
Structures of electrocatalytic nanocarbons and schemes of electrochemical devices. **a** Representative structures of the three EN types. Hydrogen fuel cell (**b**) and water electrolyzer (**c**) where EN is incorporated to accelerate the ORR, OER, and HER. A methanol fuel cell (**d**) and CO_2_ reduction cell (**e**) which make use of EN for the MOR, CO_2_RR, ORR and OER. A zinc-air battery (**f**) where EN functions as a cathode electrocatalyst for the ORR and OER. A nitrogen reduction or oxygen reduction cell (**g**) where EN is being investigated for the NRR and 2e^−^ ORR for electrochemical production of ammonia and hydrogen peroxide, respectively.

As our energy consumption continues to escalate, it is increasingly important that energy conversion and chemical production technologies progress alongside. Improving the efficiency of all involved electrochemical steps and coupling processes that require the input of energy to drive pertinent reactions with green sources, such as wind and solar power, provides a gateway to a renewable future. Inspired by this outlook and motivated by the remarkable success of the Li-ion battery technology, scientists and engineers are exploring advanced electrochemical transformations, many of which involve light, earth-abundant elements, such as C, H, N, O, and S to derive chemical conversion materials with high energy density. Heavily involved is the chemistry of oxygen, because the release of stored chemical energy is performed via the oxidation of the “fuel” using molecular oxygen. For example, molecular O_2_ is the oxidant responsible for converting the fuel (H_2_) to water and generating electricity in the hydrogen fuel cell (Fig. 1b). The reverse process, thermodynamically uphill water electrolysis generates the fuel and oxidant (Fig. 1c). Similarly, methanol is used as fuel in the methanol fuel cell (Fig. 1d), and may be regenerated via reduction of CO_2_ (Fig. 1e). Finally, some light metals, like Zn, operate as anodes in metal-air batteries (Fig. 1f). In addition to energy conversion applications, electrochemical conversions are explored for direct synthesis of industrially relevant chemicals. For example, the electrochemical reduction of oxygen or nitrogen to hydrogen peroxide or ammonia, respectively, (Fig. 1g) represents a promising alternative for the costly anthraquinone and Haber–Bosch processes currently used on large scales.

Electrocatalytic nanocarbon (EN), defined here as any form of chemically modified graphitic nanocarbon, has emerged as an excellent electrode material for the chemical transformations central to the applications described above. The last several years have witnessed an explosion of scientific studies reporting excellent performance of EN in accelerating the oxygen evolution reaction (OER)^1^, oxygen reduction reaction (ORR)^2–7^, carbon dioxide reduction reaction (CO_2_RR)^8,9^, nitrogen reduction reaction (NRR)^10,11^, hydrogen evolution reaction (HER)^12,13^, among others. Interest in EN as electrocatalysts for energy conversion and chemical production arises from four main qualities: (1) Because it is manufactured from earth-abundant elements like carbon, nitrogen, and small amounts of transition metals, it is a low-cost alternative to state-of-the-art noble metal electrocatalysts. (2) The molecular structure of EN is mostly comprised of sp^2^-hybridized, graphitic carbon, making it an excellent conductor with rapid electron transfer kinetics. (3) Synthesis of EN is amenable to a high degree of heteroatom dopants and incorporation of catalytic motifs into the molecular structure. Similar to homogeneous electrocatalysts, the active site structure may be tuned for activity towards specific reactions. (4) The hierarchical 3D structure, with regards to porosity, is easily tailorable through synthetic means, which offers large surface areas and access to an abundance of catalytic sites.

While graphitic nanocarbon is chemically inert, the catalytic activity of EN is introduced via defects and heteroatom dopants, both metal and metal-free. Generally, defects are induced through a thermal or radiation treatment of existing graphitic carbon, while dopant atoms are introduced via the reaction of a heteroatom-containing carbon precursor at elevated temperatures, i.e., pyrolysis. This review discusses three types of EN, categorized based on the nature of the chemical modifications. Catalyst-grafted electrocatalytic nanocarbon (CGEN) results when well-defined molecular catalysts are non-covalently anchored to the conductive carbon support (Fig. 1a). These hybrid materials combine the advantages of being able to tune reactivity through synthetic manipulation of molecular catalysts with improved charge transfer kinetics resulting from immobilization. Main-group electrocatalytic nanocarbon (MGEN) is prepared by a substitution of carbon with main group non-metals, e.g., B, N, O, S, or P (Fig. 1a). Here, the addition of dopant atoms introduces charge polarity into the graphitic framework, generating electron-deficient or electron-rich carbon atoms that act as catalytically active moieties. Single-atom catalysts (SACs) are macrocyclic structures in which one or two transition metal centers, such as Fe, Co, or Ni, are coordinated into the graphitic framework through nitrogen, and other, dopants that anchor metal atoms via sigma donation (Fig. 1a). Naming these structures as SACs is somewhat misleading, since their catalytic activity cannot be isolated to the transition metal center but also depends on the nature of the neighboring anchoring ligands, as well as the extended π-orbitals of the graphitic framework. However, the SAC nomenclature is used here for consistency with other literature (Fig. 1a).

Despite numerous reports of catalytic enhancements by EN, experimental challenges associated with the structural characterization of catalytic sites make it exceptionally difficult to identify the reactive centers, and understand the mechanism of their operation. As a result, a large number of studies report catalytic effects without providing additional insights into the mechanism of operation, a situation that has recently been a center of hefty criticism^14^. It seems that nearly any modification of EN can lead to an improved catalytic activity and that for the further development of EN, more detailed mechanistic studies will be a productive research direction. Although mechanistic studies may be challenging—monitoring the changes to heterogeneous solids during the catalytic cycle is non-trivial and often requires the use of expensive spectroscopies—they are necessary to inspire better understanding of the catalysis. To facilitate future fundamental research efforts, this review focuses on our current understanding of the underlying catalytic process and complements other reviews in the field of EN, which emphasize material design and characterization^15–20^. The review will begin with “Selected examples in electrocatalysis” section that describes selected examples of catalytic enhancements by EN for reactions such as OER/ORR, CO_2_RR, and others. To follow will be “Insights from spectroscopy” section which highlights the insights gained from spectroscopic studies and the inherent challenges with the used techniques. We will specifically detail the importance and difficulty of chemically selective, and in situ experiments in clearly characterizing samples and intermediates. “Insights from molecular models” section will cover mechanistic studies involving both natural systems and well-defined, biomimetic molecular models and will tie together concepts relating homogeneous and heterogeneous catalysis. “Insights from computational studies” section details computational studies, which have helped explain EN electrocatalytic effects and developed descriptors for prediction of EN activity. Finally, we will provide a summary of the covered topics and our perspective on the future research directions of EN.

## Selected examples in electrocatalysis

**Fig. 2:**
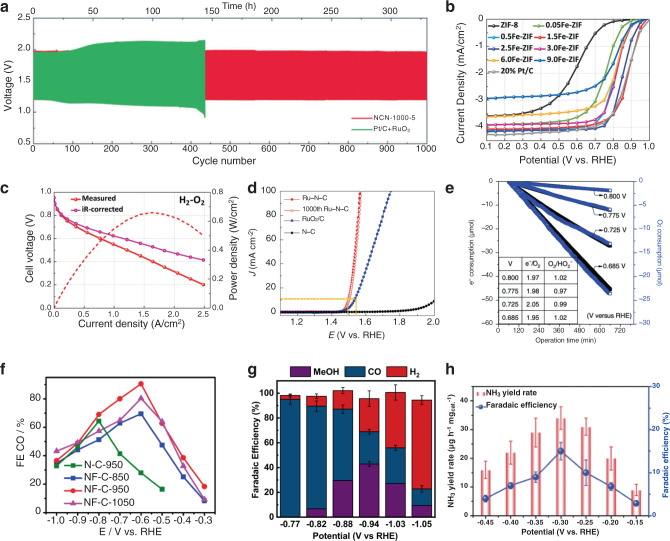
Electrochemical data from EN. **a** Galvanostatic charge/discharge cycling curves of a Zn-air battery with N-doped graphene nanosheets (red) and a Pt/C + RuO_2_ (green) cathode. Adapted and reprinted with permission from ref. ^22^. **b** RRDE polarization plots for an Fe-based SAC (red trace) where the ORR *E*_1/2_ was measured to be 0.88 V vs. RHE as compared to a Pt/C reference (gray trace) with *E*_1/2_ of 0.87 V vs. RHE. Adapted and reprinted with permission from ref. ^27^. **c** Hydrogen fuel cell performance with the same Fe-based SAC where a current density of 145 mA/cm^2^ was achieved at 0.8 V vs. RHE. Adapted and reprinted with permission from ref. ^27^. **d** Electrocatalytic OER performance for a Ru-SAC (red trace) compared with commercial RuO_2_/C reference (blue trace) in 0.5 M H_2_SO_4_. Adapted and reprinted with permission from ref. ^1^. **e** Chronoamperometry measurements toward the 2e^−^ ORR for a reduced graphene oxide electrocatalyst. The inset table shows the e^−^/O_2_ and O_2_/HO_2_^−^ measured at various potentials. Adapted and reprinted with permission from ref. ^34^. **f** FE_CO_ values measured at various overpotentials for an N and F co-doped EN (red trace). Adapted and reprinted with permission from ref. ^41^. Copyright 2019 American Chemical Society. **g** Potential dependence of FE values toward the formation of MeOH, CO, and H_2_ for the CO_2_RR and HER catalyzed by CoPC/CNT hybrid. Error bars represent one standard deviation from three measurements. Reprinted with permission from ref. ^9^. **h** Yield rates and FE values for production of NH_3_ by a Mo-based SAC. Error bars represent one standard deviation from six measurements. Adapted and reprinted with permission from ref. ^10^.

Oxygen electrochemistry is at the heart of most energy conversion strategies—aqueous systems, such as Zn-air batteries, fuel cells, and water electrolyzers, rely on the four-electron O_2_/H_2_O redox couple for their operation. From a purely thermodynamic perspective, direct four-electron reduction is desired because it, in principle, requires less energy (*E*^0^(O_2_/H_2_O) = 1.23 V vs. NHE) than processes that occur via two-electron (*E*^0^(O_2_/H_2_O_2_) = 0.77 V and *E*^0^(H_2_O_2_/H_2_O) = 1.74 V vs. NHE) or one-electron (*E*^0^(O_2_/O_2_^−^) = −0.33 V and *E*^0^ (OH^•^/H_2_O = 2.59 V vs. NHE) intermediates^21^. In practice, both the OER and ORR are met with severe kinetic barriers, leading to large overpotentials which have impeded the large-scale proliferation of Zn-air batteries, water electrolyzers and fuel cells. Due to Zn-instability in acidic solutions, Zn-air batteries utilize basic electrolytes (pH = 13). Under these conditions, MGEN has shown excellent results. For example, nitrogen-containing carbon nanotubes (NCNT) reported by Dai and coworkers demonstrated ORR half-wave potentials (*E*_1/2_) of −0.1 V vs. Ag/AgCl (0.86 V vs. RHE), rivalling that of a Pt/C reference. The NCNT was highly selective for the four-electron ORR pathway, having an electron transfer (ET) number of 3.9, unlike its non-doped CNT analog, which was selective to the two-electron pathway. Stability of the NCNT electrode was confirmed by cyclic voltammetry (CV) cycling at potentials near ORR. The NCNT showed a perfectly stable response up to 100,000 successive cycles, while the Pt/C reference showed a degradation of current. Finally, its resistance to crossover effects and catalyst poisoning were tested by exposure to methanol and carbon monoxide, respectively, near ORR potentials. Unlike the Pt/C, which showed rapid current deterioration in these conditions, NCNTs had a much more stable activity^2^. Certain MGENs have demonstrated bifunctional activity, making them even more intriguing as a Zn-air battery electrocatalyst for both the ORR and OER^3,22^. When Li et al. incorporated nitrogen-doped defective carbon nanosheets in Zn-air battery cathodes, staggering performance was achieved, relative to one with a state-of-the-art Pt/C+RuO_2_ cathode. Specifically, under fast galvanostatic discharge/charge cycles the MGEN prepared battery shows a stable discharge/charge voltage gap of ~0.8 V up to 1000 cycles, whereas the Pt/C+RuO_2_ prepared battery deteriorated to a voltage gap over 1 V within 200 cycles (Fig. 2a).^22^

Regardless of the high impact research demonstrating four-electron ORR catalysis achieved by metal-free MGEN, there has been considerable debate as to whether the active site is actually “metal-free.” Pumera et al. have argued that catalysis in supposed metal-free systems results from residual transition metal impurities (Fe, Mn, Ni, and Co), which are commonly used as catalysts for MGEN synthesis and are not adequately removed during purification^23,24^. The authors published a series of studies using commercially available graphite and carbon nanotube (CNT) samples, where highly sensitive techniques, e.g., inductively coupled plasma mass spectrometry (ICP-MS), were used to identify residual metal impurities up to 1000’s of ppm. Furthermore, catalytic activity and selectivity towards four-electron ORR corresponded to samples with higher concentrations of Mn and Fe—samples which contained no detectable metal impurity showed the worst activity towards four-electron ORR^23,24^. Counter to these efforts, Lyth, et al., have conducted multiple studies in which MGEN’s are generated via an entirely metal-free, save for Na, synthetic process with further inductively coupled plasma atomic emission spectroscopy confirming the lack of Fe impurities in samples^25,26^. Here, onset potentials of 0.85 V vs. RHE and ET numbers around 3.5 were measured for metal-free MGEN. It is noteworthy to comment that, while both sides have shown compelling evidence, the debate is not fully resolved. Studies which attributed the four-electron ORR to residual metal impurities did so via observing the loss of activity after removal of the impurities, however, the underlying carbon material came from commercial sources, where catalytic activity-imparting heteroatom dopants, were likely not present. As well, when entirely metal-free syntheses were used to prevent the presence of metal impurities, only the lack of Fe-based impurities were specifically commented on, even though it is plausible that other transition metals may have been present.

A majority of hydrogen fuel cells operate in acidic media, because they rely on efficient proton-conductive polymer membranes to deliver reactant ions to the cathode. Under these conditions, SACs appear to work as promising electrocatalysts for ORR. Initial interest in SACs for ORR was aroused by Zelenay et al., who reported Fe (PANI-Fe-C) and Co (PANI-Co-C) polyaniline-derived catalysts^4^. Here Fe-doping was identified from extended X-ray absorption fine structure (EXAFS) measurements as single atom in nature, existing as FeN_4_ moieties. The authors cited significant achievement in performance for the ORR where the half-wave potential difference (Δ*E*_1/2_), measured by rotating disk electrode (RDE), between PANI-Fe-C and a state-of-the-art Pt/C reference was only 43 mV. As well, H_2_O_2_ selectivity for PANI-Fe-C remained below 1% for all potentials^4^. Inspired by this, many successful studies on SACs for ORR have been reported^5–7^. Recently, Wu et al. were able to fabricate a metal-organic framework (MOF)-derived SAC with strong performance for ORR. The catalyst was enriched with FeN_4_ moieties by performing cation exchange to replace Zn^2+^ with Fe^3+^ prior to pyrolysis treatment. Degree and type of Fe incorporation was easily tuned by adjusting the stoichiometry of Fe dopants with the greatest performance coming from 1.5 at% which gave an ORR *E*_1/2_ of 0.88 V vs. RHE, which was more positive than the Pt/C reference electrocatalyst (Fig. 2b)^27^. Furthermore, in a practical fuel cell assembly, large current densities of 44 and 145 mA/cm^2^ were reached at 0.87 and 0.8 V vs. RHE, respectively (Fig. 2c). In the case of OER, a reaction of importance for water electrolysis systems, the most active Ru oxide-based electrocatalysts suffer from dissolution under acidic oxidative conditions. A wonderful example of stability and performance for the OER in acidic electrolyte is a Ru-based SAC published by Yao and coworkers^1^. Here, single Ru atoms are anchored to a graphitic matrix via strong Ru-N bonds in a porphyrin-like configuration. Ultimately, improvement in the OER activity, 10 mA/cm^2^ current density was reached at an OER overpotential of 267 mV, with little to no loss in activity after 1000 cycles was achieved (Fig. 2d).

One potential challenge for EN’s use in Zn-air batteries and water electrolyzers is the susceptibility of the electroactive surface area of graphitic carbons to carbon corrosion. At anodic potentials, graphitic carbons are electrochemically oxidized to CO (*E*^0^(CO/C = 0.518 V vs. SHE)) and CO_2_ (*E*^0^(CO_2_/C = 0.207 V vs. SHE)). Fortunately, these processes are kinetically slow, so no appreciable corrosion occurs at potentials required for ORR. However, exposure to prolonged oxidation results in gasification of carbon materials^28^ and the formation of passivating layers of surface oxides, affecting stability under OER conditions^29^. Although corrosion is a major concern and it should be requisite that any study, which reports a competitive anodic electrocatalyst must also investigate its electrochemical response and structural morphology pre-cycling and post-cycling, there is evidence that carbon-based OER is achievable^1^. A recent differential electrochemical mass spectrometry (DEMS) investigation showed that addition of a highly-active nickel boride OER catalyst was able to effectively suppress anodic corrosion of the carbon black support. This suggests that, assuming adequate dispersal across the catalyst support, carbon corrosion may be kinetically suppressed for other highly-active OER electrocatalysts^30^.

Besides its central role in energy conversion, the chemistry of atmospheric oxygen is responsible for production of hydrogen peroxide (H_2_O_2_), a versatile chemical with a variety of uses from industrial bleaching agent to medical disinfectant. Today’s production of H_2_O_2_ is dominated by the anthraquinone process wherein a hydroquinone is generated through Pd-catalyzed hydrogenation followed by subsequent oxidation which produces H_2_O_2_^31^_._ As it is implemented today, the anthraquinone process is considered a centralized, batch process, meaning its production is carried out at large, industrial installations. Added distillation and transportation steps make the process expensive and energy intensive. An attractive approach for decentralizing H_2_O_2_ production is the two-electron ORR which may be coupled with renewable energy sources^32,33^. To achieve this goal, significant attention has been paid to developing ENs with high selectivity towards two-electron ORR and stability under operating conditions. Encouraged by anthraquinone electrochemistry, several teams have explored the two-electron ORR using EN enriched with O-atoms and found excellent catalytic selectivity and efficiency^34–36^. For example, McCloskey used a mild reduction of graphene oxide to generate EN material with oxygen groups, that convert oxygen to hydrogen peroxide at low onset overpotential (10 mV) and high selectivities across an entire potential range (100% Faradaic efficiency, Fig. 2e)^34^. While epoxide functional groups were identified as the catalytically active moiety in this study (using in situ Raman spectroscopy), recent reports indicate that carbonyl groups are more catalytically active toward two-electron ORR^36^. First-row transition metal based SAC catalysts have also shown excellent performance in electrocatalytic H_2_O_2_ formation^37–39^. These studies point to an important role that the SAC coordination environment plays, where presence of O was found to have large effect on the product selectivity. For example, Zhang et al, synthesized a Ni-SAC with mixed N and O coordination sites based upon the molecular Jacobsen’s catalyst with NiN_2_O_2_ coordination^37^. Activity towards two-electron ORR was benchmarked against an analogous Ni-based SAC with only nitrogen atoms in the coordination environment (NiN_4_). As measured by ring current on the rotating ring disk electrode, the oxygen-containing NiN_2_O_2_ catalyst showed much improved selectivity toward two-electron ORR (96%) compared with the all-nitrogen NiN_4_ (62%). The likely role of oxygen atoms in the coordination sphere is to tune active site oxophilicity, such that adsorbed *OOH intermediates are preferentially protonated at the proximal O, rather than distal, preventing O–O bond cleavage. The presence of oxygen itself, as either a first or second shell ligand, can have a major effect on active site oxophilicity in SACs^37–39^.

The electrochemical CO_2_ reduction reaction (CO_2_RR) presents a convenient opportunity to help decelerate rising atmospheric greenhouse gas levels. Coupling this process with renewable energy sources presents a sustainable method of recycling atmospheric CO_2_ for production of valuable industrial chemicals and fuels. Most commonly, CO_2_RR proceeds through a 2e^−^ process to afford CO, an important feedstock for further conversion into organic fuels and chemicals. Heteroatom doping of graphitic carbon induces significant charge or spin redistribution and significant electronic structure modification, making them an impressive platform to improve CO_2_RR selectivity towards CO. For example, graphene foams with N-defect sites were found to perform CO_2_RR electrocatalysis with maximum Faradaic efficiency for CO (FE_CO_) of 85% at −0.58 V vs. RHE. X-ray photoelectron spectroscopy (XPS) and electrochemical data revealed that samples with the highest pyridinic-N content displayed the highest FE_CO_ values and at lowest overpotentials. Computational evidence agreed with these results, revealing the active sites to be electronegative, pyridinic-N sites^40^. Similarly, N and F dual-doped holey graphene achieved an FE_CO_ of 90% at −0.6 V vs. RHE in CO_2_-saturated 0.1 M KHCO_3_ (Fig. 2f).^41^. The holey graphene, being rich with edge sites, enabled a high density of CO_2_RR-active pyridinic sites. Nearby F co-dopants provided two important functionalities to holey graphene. First, they modulated *H binding strength of pyridinic N sites, which suppressed the competitive HER process. Second, DFT results showed that F-modified pyridinic sites possessed larger charge densities, which improved its ability to facilitate charge transfer to adsorbed *COOH intermediates.

Some more desirable CO_2_RR pathways lead directly to highly reduced end products like methanol, for example. Although CO_2_RR to methanol (*E*^0^(CO_2_/CH_3_OH) = −0.38 V vs. NHE) is more thermodynamically favorable than CO (*E*^0^(CO_2_/CO) = −0. 53 V vs. NHE), there are still very few examples which cite it as a product. Instead, most findings suggest that metal-free MGEN and macrocyclic SACs are capable of producing CO at high FEs and suppressing the competitive HER^40–48^. However, recent work by Wang’s group has realized some surprising results and perhaps discovered a promising platform by which one can invert the selectivity of CO_2_RR. The authors investigated a series of functionalized cobalt phthalocyanines (CoPC) immobilized on conductive CNT supports and found significant methanol production (44% FE_MeOH_ at −0.94 V vs. RHE, Fig. 2g) proceeding through an initial two-electron reduction to form the crucial *CO intermediate, followed by a cascading series of PCET steps to generate MeOH^9,49^. The authors observed production of methanol began to wane during long-term electrolysis because of the undesired reduction/protonation of pyrrolic nitrogen in CoPc/CNT. To circumvent this issue, the authors modified the CoPc unit with electron-donating amine groups, effectively shifting the reduction potential to more negative values and achieved a stable MeOH FE at −1.0 V vs. RHE^9^. Some initial explanations for MeOH production hinted at improvements to catalyst diffusion via immobilization, which promoted the transference of multiple electrons to adsorbed intermediates. However, this seems unlikely, as SACs share a similarly immobilized active site, but no selectivity inversion is observed for SACs or covalently-linked molecular electrocatalysts^42–47^. The mechanisms behind improved methanol selectivity are still not known, but there have been follow-up studies tasked with unraveling these findings^50,51^. A recent study by Wang et al., was able to confirm that CO_2_RR catalysis by molecular CoPC is actually controlled by electrochemically adsorbed, or heterogenized, CoPC molecules on the electrode surface. Furthermore, they observed a direct relationship between FE_MeOH_ and lower mass loadings of CoPC which suggests that the greater degree of CoPC dispersion, lowest aggregation and best contact with conductive electrodes, leads to the highest FE_MeOH_^51^. It appears, then, that the change in CO_2_RR selectivity has not resulted solely from catalyst diffusion improvements, but also from the high degree of electronic communication between single CoPC sites and the conductive CNT/electrode.

During the last century, the Haber–Bosch process has been relied upon for production of NH_3_, an important precursor for our agricultural needs, and accounts for 1–2% of global energy consumption. While nitrogen hydrogentation is an exothermic process (N_2_ + 3H_2_ → 2NH_3_, Δ*H* = −21.9 kcal/mol), the high temperatures required to avoid catalyst poisoning by ammonia make it an energetically demanding process^52,53^. An electrochemical approach involving NRR at the cathode and OER at the anode, is equally demanding^54^. Despite similar energy requirements, the ability to drive the process using electrochemical methods is advantageous, as it is expected to enable successful coupling of ammonia production with renewable energy sources and circumvent the need for reaction with H_2_, which is currently formed using fossil fuels. The challenges associated with NRR are two-fold: (i) HER occurs in competition: the standard reduction potentials for the two processes are similar, while HER is less kinetically challenging than NRR, making it difficult to avoid; (ii) kinetics of the reaction are slow, due to barriers associated with activation of chemically inert N_2_ molecules, thus, catalysis is needed. Influenced by natural, nitrogenase systems which feature a Mo-based active center, Xin synthesized a Mo-SAC for the NRR^10^. Maximum ammonia yield rates of 34.0 μg/cm^2^ h, and FEs of 14.6% were achieved at −0.3 V vs. RHE (Fig. 2h). Activity towards the NRR was ascribed to atomically dispersed Mo sites, which have been computationally shown to stabilize and destabilize the crucial *N_2_H and *NH_2_ intermediates, respectively.

Detection of ammonia and attribution of its production to NRR have proved problematic. Reported yield rates for NRR are still low and, in some cases, ammonia present from electrochemical processes and exogenous sources, e.g., air or labile nitrogen functional groups, may be indistinguishable. The need for implementation of methods which purify feed gases, e.g., Ar and N_2_, and unambiguously assign ammonia to NRR are evident. Two protocols for rigorous benchmarking of NRR electrocatalysts have recently been reported^54,55^. In effect, they each may be broken down into five steps: 1) Purification of feed gases which may contain exogenous NH_3_ and oxides which are easily reduced to NH_3_ (NO_x_); 2) Control experiments with both Ar and N_2_ performed at open circuit potential which quantify amounts of ammonia native to the reaction system; 3) Electrochemical reduction of N_2_ using ^15^N isotopically-labelled feedgas and quantification with isotope-sensitive methods, e.g., NMR where the ^15^NH_4_^+^ and ^14^NH_4_^+^^1^H peaks are resolved into two and three symmetric signals, respectively. Importantly, the amount of NH_3_ produced in ^15^N and ^14^N reduction tests must be in agreement; 4) Finally, experiments which show NRR reduction must be repeated for reproducibility and statistical consequence with important electrochemical values uniformly reported, e.g., geometric area normalized ammonia yield rate (mol/cm^2^ s).

## Insights from spectroscopy

Confident identification and interpretation of EN is difficult due to several factors. First, EN is composed of graphitic C-atoms that contain numerous vacancies and defects, while catalytic sites are distributed unevenly across the materials. This amorphous nature of EN and lack of long-range order prevents the use of X-ray diffraction methods for their characterization. Second, the heterogenous element usually presents itself at dopant levels, requiring characterization techniques to have high chemical sensitivity to EN. Finally, coupled with these two problems is the chemical variability among even the same dopant elements (such as pyridinic, pyrollic, and graphitic N-groups). Accordingly, only a few techniques can be used to confidently identify and interpret changes to EN. Core spectroscopies, such as XPS and X-ray absorption spectroscopy (XAS), have been heavily used for elemental identification and characterization of its bonding environment and local structure. The element-specificity of XPS and XAS enable selective identification of non-metal and metal-based heteroatoms, such as N and Fe doped into the nanocarbon framework, while the development of bright synchrotron X-ray sources has enabled low limits of detection needed to identify small amounts of heteroatoms. Furthermore, these techniques are sensitive to the type of functional groups that are formed between non-metal heteroatoms and carbon, as well as oxidation states and coordination environments of metal centers. Another useful technique, albeit limited to a small number of elements, like Fe, is Mössbauer spectroscopy, which is capable of identifying a probed metal centers’ bonding environment and distinguishing alternative coordination environments.

**Fig. 3:**
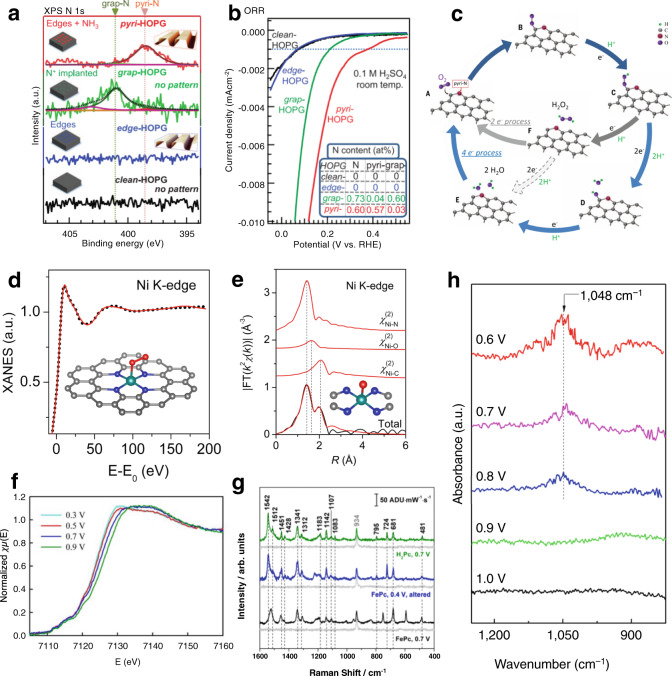
Spectroscopic data from EN studies. **a** N 1*s* XPS spectra, and **b** linear sweep voltammograms of HOPG electrodes functionalized with pyridinic (red) and quaternary (green) nitrogen and unfunctionalized edge-plane (blue) and cleaned basal plane (black) HOPG. Adapted and reprinted with permission from ref. ^58^. **c** The proposed reaction mechanism where O_2_ adsorbs onto the carbon adjacent to the pyridinic nitrogen to form structure B, which then undergoes a series of PCET steps to form either H_2_O_2_ or H_2_O^58^. **d** Theoretical fitting (red lines) of EXAFS spectra for an Ni-doped SAC with O_2_ adsorbed on the metal site. Reproduced with permission from ref. ^63^. **e** Ni K-edge FT-EXAFS belonging to a Ni-SAC showing the experimental spectra (black) and best fit (red) from the inset model along with contributions from, top to bottom, Ni–N, Ni–O, and Ni–C scattering. Reproduced with permission from ref. ^63^. **f** In situ Fe K-edge XANES of an Fe-SAC at anodic potentials approaching and surpassing the onset of ORR. Adapted and reprinted with permission from ref. ^62^. **g** In situ Raman of FePC molecules deposited on a gold TERS substrate working electrode at 0.7 V (unaltered, bottom plot) and 0.4 V (altered, middle plot) with H_2_Pc at 0.7 V for comparison. Adapted and reprinted with permission from ref. ^71^. **h** Operando SR-FTIR measurements recorded during ORR for a NiFe-co-doped MOF. A potential-dependent band arises at 1048 cm^−1^ and continues to grow as potentials become more negative. Adapted and reprinted with permission from ref. ^70^.

XPS probes the energy required to eject an electron from core orbitals of specific elements present in the sample, revealing detailed chemical environment information. For example, nitrogen atoms with increasing partial positive charge are more difficult to ionize and thus exhibit higher binding energy (BE) in XPS spectra. Based on this argument, the order of N 1*s* binding energies can readily be predicted as follows: pyridinic<graphitic<pyrrolic~pyridonic (the resolution of XPS is insufficient to resolve pyrrolic and pyridonic groups). This trend has been observed in the XPS spectra of numerous small organic molecules that contain relevant nitrogen functionalities^56^, and these studies serve as benchmarks for assignment of nitrogen functionalities in EN, and are supported by DFT calculations^57^. Pyrolytic methods generate a range of *N* functional groups at different proportions and as a result there has been large debate about which functional group is responsible for ORR activity^58^. XPS analysis has been critical in settling the debate and identifying the responsible group. To provide insight into the mechanism, Nakamura and co-workers performed controlled synthesis of pyridinic and graphitic carbon electrodes with use of highly oriented pyrolytic graphite (HOPG) substrates. XPS spectra in Fig. 3a show that their synthetic procedure produced pure pyridinic and graphitic HOPGs with BEs of 398.5 and 401.1 eV, respectively. The authors unambiguously showed that pyridinic N exhibit superior catalytic performance, as illustrated with lower ORR overpotentials observed in their voltammograms (Fig. 3b). XPS measurements after ORR cycling showed the presence of pyridonic groups, which led them to propose the ORR mechanism presented in Fig. 3c. The mechanism involves adsorption of O_2_ onto the carbon adjacent to pyridinic N (Fig. 3c: A→B). Upon one-electron reduction, a hydroperoxide is formed (B→C), which represents an important branching point between the undesired two-electron (H_2_O_2_, C→F→A) and four-electron pathway to water (C→D→E→A) that results in formation of pyridonic intermediate D detected in the XPS.

XAS, a combination of X-ray absorption near-edge structure (XANES) and EXAFS, is an extremely useful technique for analyzing the nature of catalyst sites. In XANES, edge positions generally provide information on the oxidation state of the absorber center, while pre-edge features can be used to analyze bonding environments around the absorber atom (deviation from centrosymmetricity will yield an increase in pre-edge intensity)^59^. EXAFS can probe local structure of the first coordination shell and beyond. Specifically, fitting Fourier transform-EXAFS provides information about shell distances between absorber and scattering atoms and coordination number of the absorber^59^, while Wavelet transform-EXAFS can distinguish between different backscatterers in the same shell^60^. L-edge XANES tells about oxidation state and bond covalency of a metal^61^, while the L_3_/L_2_ intensity ratio indicates a metals’ spin state and has been used to clarify results of other techniques, such as Mössbauer spectroscopy^62^. All these methods stemming from XAS can be used in combination to achieve a clear picture of the catalytic environment. For example, a recent XAS study of several first-row transition metal SACs for OER has shown that all metals form identical MN_4_C_4_ geometry within the graphene framework^63^. Figure 3e shows the EXAFS curve fitting for Ni-based SAC sample, showing three distinct backscattering contributions: Ni–N and Ni–O bonds give rise to the peak at 1.44 Å, while Ni–C scattering contributes to the peak at 2.01 Å. Subsequent structural refinement of experimental XANES profiles of Fe, Ni, and Co-based SACs (Fig. 3d), revealed that all three SACs exhibit identical geometry in which the transition metal is surrounded with four in-plane nitrogen atoms, and one adsorbed oxygen atom in the first shell. The second coordination shell is composed of four C-atoms, as shown in the inset structures.

Mössbauer spectroscopy is useful for characterization of SAC sites involving a limited number of nuclei (most notably ^57^Fe), where three main spectral features contribute to structural information about the material^64^. Firstly, an overall offset from zero (reference) in the spectrum is known as an isomer shift, and it provides information about oxidation state of the element. Secondly, quadrupole splitting occurs due to asymmetric electron distribution around the probed nucleus. In the case of ^57^Fe, quadrupole splitting causes formation of a doublet in the spectrum, which is indicative of single Fe sites, while the presence of sextet and singlet components is attributed to crystalline phases of Fe^65^. Thirdly, in the presence of an external magnetic field, additional hyperfine splitting occurs due to the Zeeman effect. In the field of EN, Mössbauer spectroscopy has been particularly suitable as a method for distinguishing between different Fe-based functionalities within one heterogeneous sample, including different SAC sites, Fe-carbide, and metallic Fe. The doublet associated with SAC sites, often labeled as D1 and D2, have been difficult to assign, due to absence of appropriate reference compounds^66^. Recent computational work has shown that the D1 doublet is consistent with FeN_4_C_10_ motifs identified in XAS studies, and further show that Fe is most likely present in its high spin (*S* = 5/2) Fe^3+^ state^67^. Doublet D2 has been assigned to Fe(II)N_4_C_10_ low (*S* = 1) spin state^67^.

Answering the question of how the active site changes during electrochemical processes is highly important towards achieving a rational design of future catalyst. In situ measurements that probe the system spectroscopically over the course of operation ideally offer researchers a view into the catalyst dynamics. As catalytic processes are very quick in nature, in situ studies mostly involve monitoring stable intermediate states as a function of applied potential, but do not identify shorter-lived transient states. For example, in situ XANES measurements at the Fe K-edge of MOF-derived SACs embedded in porous carbon exhibited an edge shift towards higher energy with increasing applied voltage. The authors attributed this edge shift to the transition of Fe^2+^ to Fe^3+^ (Fig. 3f). When reasoned in combination with a high L_3_/L_2_ (>3) ratio from L-edge XANES, the authors suggested that the high-spin O_x_Fe^3+^-N_4_ was reduced to HO^−*^Fe^(2+)^-N_4_ during ORR operation^62,68^.

Synchrotron radiation Fourier-transform infrared spectroscopy (SR-FTIR) has recently emerged as a very useful technique for mechanistic studies involving heterogeneous electrocatalysts. Development of high intensity IR beamlines has made identification of some insensitive, adsorbed intermediates possible. For example, a Co SAC with activity towards OER was studied in situ by SR-FTIR. At potentials near the OER onset, authors observed a potential-dependent red shift of the Co–N stretching band at 1048 cm^−1^ and subsequent growth of a new band at 1248 cm^−1^, which resulted from Co–O stretching. The authors asserted that the Co–N stretch red shift resulted from a potential-driven electron transfer from Co to N and promoted formation of key *O intermediates^69^. In a separate study on a NiFe-co-doped MOF with activity towards ORR and OER, investigators witnessed evolution of another potential-dependent band at 1048 cm^−1^ (Fig. 3h). Pairing their tentative assignment of the band to the adsorbed superoxide intermediate (*OOH) with operando Ni L-edge data, they saw that coincident with evolution of *OOH IR bands was emergence of a Ni^4+^ state, leading to a converging explanation that the Ni^4+^ state is the active site responsible for oxygen electrocatalysis^70^.

Using molecular catalysts as test cases is also instructive for in situ studies as the type of catalytic site can be directly selected for probing. One example used Fe-phthalocyanine (FePC) molecules to investigate the mechanism of well-known dissolution of SAC metal species into electrolyte, rendering the catalyst deactivated. To this end, in situ tip-enhanced Raman spectroscopy (TERS) was used to identify the presence of demetalated Pc after ORR^71^. The authors found existence of a significant change in the Raman shift of 1524 to 1542 cm^−1^ (Fig. 3g), which was attributed to the change in cavity size of Pc from (FePc→H_2_Pc). The authors concluded that metal site degradation could occur below carbon corrosion potential, and can be an independent phenomenon. Another application of molecular forms of catalytic sites comes from electrochemical scanning tunneling microscopy (ESTM) of CoPc under CO_2_ atmosphere. The researchers observed increases in electron density over deposited CoPc monolayers only when CO_2_ is introduced into the system and when potential is applied^72^. By sweeping the potential cathodically, the site density increased. Reaction rate constants were estimated by counting the evolution in number of high electron density units from the time of potential application to equilibrium.

In summary, the nature of electrocatalytic sites in EN has been proposed based on data obtained using element-sensitive spectroscopic techniques. However, it is important to emphasize that no one spectroscopic technique offers a complete view of EN structure. For example, it was recently demonstrated that the EXAFS of some supported CuO and NiO nanoparticles was not sensitive enough to fully distinguish them from SACs^73^. As such, it is important to utilize multiple spectroscopies, each providing complementary information about the metal centers and the associated coordination sphere. It is also essential that in situ electrochemical studies are reported, since these studies provide important mechanistic insights. When used in situ, changes to EXAFS and XANES spectra can be representative of structural distortions and changes in active site electronic structure, consequences of active site, and substrate interaction/hybridization. Similarly, spectroscopies like FTIR and Raman are sensitive to the vibrational modes of a sample and can thus monitor interactions between the substrate, active site, and support. Coupling together a full suite of ex situ and in situ measurements can result in confident assignment of active site geometric structure along with the observance of some intermediate states.

## Insights from molecular models

**Fig. 4:**
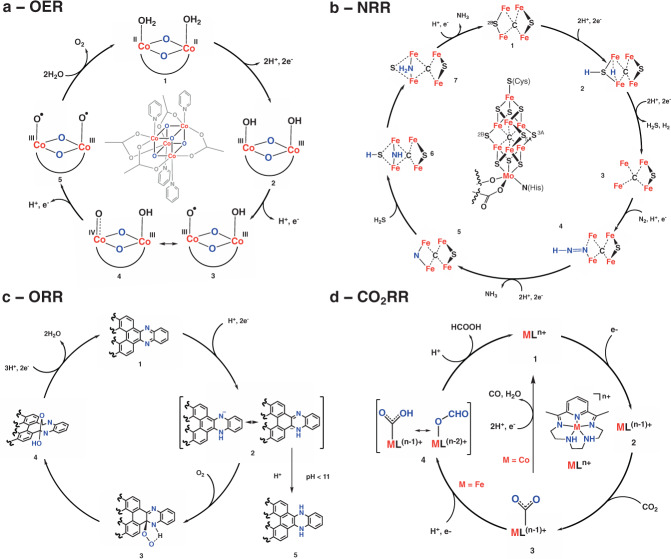
Catalytic mechanisms deduced from molecular models. **a** Co-cubane OER catalysts and its catalytic mechanism. Catalyst undergoes four-electron oxidation shared between two Co-centers yielding an oxyl intermediate 5 which then forms O–O bond^75^. **b** NRR asymmetric mechanism considered as a possible pathway for nitrogenases^78^. **c** ORR mechanism accepted for metal-free GQDs showing carbanion (3) intermediate which is crucial for O_2_ activation. However, if pH is lowered, protonation becomes fast yielding intermediate 5 and shutting off the catalysis^79^. **d** CO_2_RR mechanism showing branching between CO and formate-forming cycles from intermediate 3. Transition metal used in complex determines the path catalysis undertakes^85^.

In parallel with research on catalytically active EN materials, molecularly precise models have been extensively studied as homogeneous electrocatalysts for OER/ORR/CO_2_RR/NRR. Several advantages make them ideal systems for mechanistic studies: well-defined structures that are characterized with high fidelity, structural tunability that enables optimization of catalytic activity through rational design of active species, and accessibility of techniques to study catalytic mechanisms. Insights gained from those studies are envisioned to guide design of future catalytic materials. One of the most important insights from these studies is that catalytic motifs are never single atoms. For example, multielectron chemistry, such as OER, is challenging for a single metal catalytic site to accommodate. While late precious transition metals, such as Ru, can support such large oxidation state changes (from 2+ to 5+)^74^, earth-abundant, first row transition metals, such as Co, usually undergo only one or two-electron oxidation. Thus, OER often involves bimetallic sites, such as the Co-cubane model presented in (Fig. 4a)^75^. Extensive spectroscopic and electrochemical studies of Co-cubane showed that two Co active sites undergo a sequence of PCET steps to form intermediate structures 2–5 (Fig. 4a), followed by the rate-determining O–O bond formation step from intermediate 5. Such bimetallic catalysis is believed to occur in many OER catalysts, such as enzymatic Mn-based clusters^76^ and artificial transition metal oxide catalysts^77^, confirming the importance of cooperative effects between multimetallic sites in electrocatalysis.

In addition to cooperativity of multiple metal centers, the best catalyst performance requires synergism between ligands. First and second coordination spheres play important roles in catalytic cycles such as providing pathways for protons, electrons, substrates, and products or adjusting local pH to the required value for particular catalytic steps. A beautiful example illustrating ligand participation can be found in enzymatic NRR involving Mo-nitrogenase. While the exact catalytic pathway is still not known, a plausible mechanism that is consistent with experimental evidence^78^ and computational studies^53^ is presented in Fig. 4b. The catalyst consists of a large FeMo cofactor, whose role is to facilitate accumulation of electrons and protons needed for reduction, provide a binding site for dinitrogen and reduce it to two molecules of ammonia. Interestingly, the enzymatic reaction involves eight PCET steps (only six are necessary for NRR), where the additional two PCET processes are needed for the release of H_2_ and H_2_S needed to generate coordination vacancies at Fe-centers needed for N_2_ binding and activation (Steps 1→3, Fig. 4b, the structures show the replacement of one of the sulfur belt atoms with N_2_)^78^. Subsequent PCET processes generate NH_3_ molecules, whose release is coupled with the return of the sulfur belt atom to the Fe-centers (Steps 4→1). This dynamic Fe–S metal–ligand equilibrium represent an important mechanism of providing highly reactive vacancies for substrate binding.

One feature in common to all aqueous electrocatalysis is the PCET mechanism. Transferring protons simultaneously with electrons avoids charge buildup, and therefore circumvents formation of high energy intermediates. A simple inspection of the four mechanisms presented in Fig. 4 illustrates the importance of PCET by showing that most electrons are transferred in concert with protons. However, in some cases PCET can prevent desired reactivity. Li and coworkers^79^ studied well-defined N-containing graphene quantum dots as models for ORR catalysis by N-doped nanocarbon (Fig. 4c). The authors observed that ORR catalysis occurs only at pH > 11, in agreement with many reports of efficient ORR by N-doped MGEN in basic media^3^. Based on the Pourbaix diagrams, the authors identify anionic intermediate 2 as the key intermediate activating molecular oxygen towards reduction to hydroperoxide 3. Reactive sites are identified as carbon atoms adjacent to nitrogen atoms, which is in a good agreement with ORR studies on analogous metal-free models^80–83^. Subsequent computational investigation of the catalytic cycle^84^ identified an unusual ether-like structure 4 as an intermediate, that ensures the four-electron pathway. The lack of catalytic activity at pH < 11 was assigned to fast protonation following two-electron one-proton PCET to form neutral intermediate 5, which is not sufficiently reactive to activate O_2_.

Product selectivity is one of the most challenging issues in electrocatalysis, particularly in the case of CO_2_RR where many products can be formed, such as CO, H_2_, formate, oxalate, methanol, etc. Selectivity toward one of the products is dictated by the energies of relevant metal-substrate adducts. For example, a recent study involving Co and Fe complexes 1 and 2 (Fig. 4d) showed how strength of C–O bonds in M–COOH intermediates control the product branching pathways^85^. Specifically, the authors found that Co-complex 1 reduces CO_2_ to CO, while Fe-complex 2 selectively forms HCOO^−^. Their mechanistic studies reveal that both complexes, upon two-electron reduction, react with CO_2_ through the carbon atom to form intermediate 3 (Fig. 4d)^85^. However, electron-rich Co(II) destabilizes the C-O bond through backbonding, leading to its breakage and formation of CO and H_2_O. Since Fe-complex 2 is a poor π-donor, intermediate 4 isomerizes into the O-bonded derivative, which leads to formation of the formate ion^85^. Product selectivity is also an important issue in ORR, where four-electron (H_2_O) and two-electron (H_2_O_2_) products are observed. For example, ORR catalyzed by Fe-porphyrins proceeds through a peroxo intermediate, the key structure for H_2_O/H_2_O_2_ product selectivity. If the distal oxygen is protonated a second time, the O–O bond will be cleaved and consequently four-electron reduction to water occurs. If, instead, the proximal oxygen is protonated, the O–O bond will be conserved, leading to predominantly H_2_O_2_ formation^86,87^. While Fe-based macrocycles could give both two-electron and four-electron reduced products, mononuclear Co-based macrocyclic catalysts usually selectively produce H_2_O_2_. A recent study by the Stahl group has shown that protonation of proximal oxygen is responsible for H_2_O_2_ formation, similar to Fe-based catalysts^88^.

**Fig. 5:**
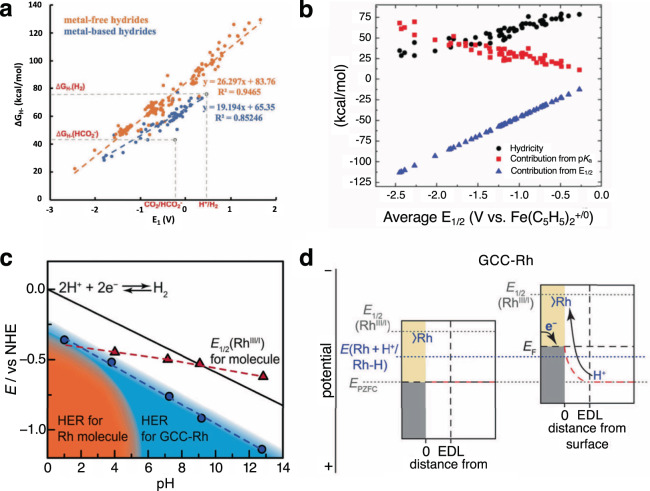
Thermodynamic properties of molecular catalysts. **a** The scaling relationships between the thermodynamic hydricity (Δ*G*) and the first reduction potential *E*_1_ of the metal-free (orange) and metal-based hydrides (blue). Adapted and reprinted with permission from ref. ^89^. **b** Two-electron redox potential (blue triangles) and pK_a_ (red squares) contributions to Δ*G* (black circles) of metal hydrides plotted by their average redox potentials. Adapted and reprinted with permission from ref. ^92^. **c** Pourbaix diagram for Rh molecular catalysts (red triangles) and Rh attached to glassy carbon electrode (GCC-Rh, blue circles). The black solid line denotes the thermodynamic potential of HER. The orange region denotes the potential-pH region in which the model is catalytically active, while the blue region shows conditions where GCC-Rh catalyzes HER^97^. **d** Proposed interfacial free energy diagrams for GCC-Rh electrodes. The gray area represents the filled band states of the electrode, while beige denotes the unfilled band states. The dotted horizontal black line indicates the *E*_F_ of the electrode. The electrical double layer, EDL, is labeled by a vertical dotted black line. The redox potential of the molecule (*E*_1/2_(Rh^III/I^)) and the potential for formation of a Rh−H species at the GCC site (E(Rh + H^+^/Rh−H)) are illustrated with dotted gray and blue lines, respectively. The electrostatic potential is shown with the dotted red line, and the potential of zero free charge (EPZFC), is indicated with a dotted gray line. Adapted and reprinted with permission from ref. ^97^, https://pubs.acs.org/doi/full/10.1021/jacs.9b04981. All future permission should be directed to the ACS.

One of the key parameters that determines catalytic performance is the overpotential. Intuition tells us that catalyst reduction potential is directly related to its reactivity: if a more negative potential is needed for catalyst reduction, the reduced form will be more reactive. Indeed, this scaling relationship has been observed in many systems, including hydride-based catalysts for CO_2_RR and HER (Fig. 5a), where standard reduction potentials associated with hydride generation scale linearly with their hydricities^89–91^. Importantly, this scaling relationship dictates the minimum overpotential that a catalyst can achieve: if the line passes through the point with zero overpotential (as is the case for HER catalysis by metal-based hydrides, Fig. 5a), chemical tuning of the structure will enable discovery of an ideal catalyst. If, on the other hand, the line does not cross the point of zero overpotential (as is the case for formate formation, Fig. 5a), even the best catalyst will exhibit an overpotential. That minimum overpotential is 1.1 V for metal-based hydrides and 1.5 V for metal-free hydrides (Fig. 5a). Discovering ways of breaking such scaling relationships is a crucial aspect of catalysis research and often requires partitioning the catalyst into separate chemical units that can be tuned independently. For example, the overall hydride transfer needed for formate formation can be achieved using a catalyst composed of separate electron and proton sources. Since reduction potentials and pK_a_ values exhibit different scaling relationships (Fig. 5b)^92^, an ideal catalyst with zero overpotential can be more readily discovered by independently tuning the co-catalytic units.

While the fields of homogeneous and heterogeneous electrocatalysis have been advancing on parallel tracks, there is still a large knowledge gap between them—it is not known what fundamentals govern the heterogenous inner-sphere electron transfer (ISET) nor how mechanisms of electrocatalysis change once molecular catalysts are placed in strong electronic communication with conductive electrodes. Recent studies indicate that interesting changes in faradaic efficiencies and product selectivity can take place. For example, immobilization of Co-phthalocyanine onto a conductive CNT support yielded reduction of CO_2_ to methanol in comparison to two electron reduction to CO for the solution-phase molecule^9^. The authors attribute this change in reactivity to restricted diffusion of the adsorbed catalyst: unlike the homogeneous model, the immobilized catalyst cannot diffuse away from the electrode surface, which enables multielectron transfers needed for formation of methanol^9^. In addition to immobilization effects, adsorption of the catalyst to the carbon support alters its electronic properties, as was observed in Fe-based ORR catalysts^93^. Specifically, it was shown that the incorporation of molecular Fe-phthalocyanine catalysts onto graphitic supports using pyrolytic methods improves ORR performance due to the electron-withdrawing effect of the carbon pi-system^93^. There has been an avenue of studies exploring larger conjugated structures, graphene nanoribbons (GNR), with Re catalysts grafted onto edges. It has been shown that this extended conjugation lowers the overpotential for CO_2_RR to CO compared to smaller models^94^. Surprisingly, the metal experienced no oxidation state change in the process and the overall catalytic reaction was described, as a transfer of electron density from a two-electron reduced ligand to CO_2_^95^. Similar phenomena was observed when Re catalysts were embedded on the edges of glassy carbon electrodes^96^.

The Surendranath group has explored molecular catalysts grafted to edge-planes of carbon electrodes and discovered significant changes in PCET behavior^97,98^. For example, Rh-based HER catalysts grafted to carbon electrodes, exhibit catalytic behaviors over a full pH = 0–14 region, in stark contrast to the homogeneous catalyst, which operates only in the acidic region (Fig. 5c). These results were rationalized as follows: the molecular model undergoes PCET and efficient catalysis in the acidic region, while catalysis is prevented in the basic region by electron transfer processes that are not coupled with proton transfer. The grafted catalyst, on the other hand, is in strong electronic communication with the conductive electrode, which prevents undesired “non-coupled” electron transfer processes from taking place. Instead, PCET proceeds in the entire pH region, controlled thermodynamically by the point of zero free charge of the electrode (Fig. 5d). Similarly, it has recently been shown that strong coupling between glassy carbon electrodes can be achieved through basal plane modification with nitrogen-doped GNRs. When the GNRs are determined to be in strong communication with the electrode surface, every ET across the entire pH region (0–14) was coupled to PT^99^. These results point to an important difference between homogeneous and heterogeneous catalysts: while isolated electron transfer events can take place in homogeneous catalysts, they are always coupled by a compensating transfer of charged groups, e.g., protons, in the case of heterogeneous catalysis.

## Insights from computational studies

**Fig. 6:**
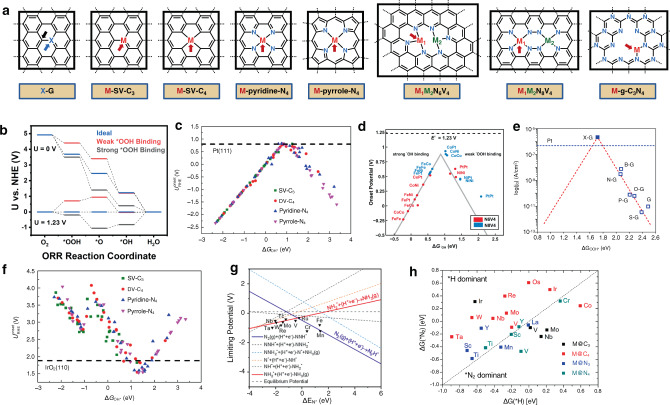
Volcano relationships from EN computational studies. **a** EN cells which have been investigated in computational studies. **b** Thermodynamic plot which shows the free energy of each elemental step for ideal (blue) and non-ideal (red and gray) catalysts for the ORR at O and 1.23 V applied potential. **c**, **d** ORR volcano plots for single-SACs and bimetallic-SACs, showing the theoretical onset potentials (${U}_{{\rm{RHE}}}^{{\rm{onset}}}$) versus OH adsorption free energies (${\triangle G}_{{\ast }_{{\rm{OH}}}}$). Adapted and reprinted with permission from refs. ^108,109^. **e** ORR volcano plot for MGEN constructed from plotting the theoretical exchange current density (${j}_{0}^{{\rm{theory}}}$) versus free energies of OOH adsorption (${\triangle G}_{{\ast }_{{\rm{OOH}}}}$). Adapted and reprinted with permission from ref. ^110^, https://pubs.acs.org/doi/10.1021/ja500432h, all future permissions should be directed to the ACS. **f** OER volcano plot for single-metallic SACs, showing the theoretical onset potentials (${U}_{{\rm{RHE}}}^{{\rm{onset}}}$) versus OH adsorption free energy (${\triangle G}_{{\ast }_{{\rm{OH}}}}$). Adapted and reprinted with permission from ref. ^108^. **g** NRR volcano plot for C_3_N_4_ SACs constructed from plotting theoretical limiting potentials versus free energy of N adsorption (${\triangle G}_{{\ast }_{{\rm{N}}}}$). Adapted and reprinted with permission from ref. ^111^. **h** Selectivity plot for the NRR vs. HER for SACs constructed by plotting free energy of N adsorption (${\triangle G}_{{\ast }_{{\rm{N}}}}$) versus free energy of H adsorption (${\triangle G}_{{\ast }_{{\rm{H}}}}$). Adapted and reprinted with permission from ref. ^112^.

Computational density functional theory (DFT) studies of electrocatalytic processes were pioneered by Norskov and co-workers in their studies of ORR catalysis by transition metal and oxide surfaces^100^, and have been applied to EN, like those shown in Fig. 6a. In their approach, an overall n-electron reduction or oxidation reaction is divided into n proton-coupled one-electron transfer processes, each resulting in formation/breakage of key reaction intermediates at the electrode surface (such as *OH, *O, and *OOH in OER/ORR, Fig. 6b). Instead of calculating Gibb’s free energies of protons and electrons, the method relates these to the calculated free energy of molecular hydrogen using the electrochemical potential of the standard hydrogen electrode (SHE, H^+^ + e^−^ $\rightleftarrows$ 1/2H_2_). Reaction overpotential is calculated by adding free energies for individual steps, leading to a simple computational method that enables screening large numbers of model catalysts. Even though this purely thermodynamic model neglects kinetic barriers associated with, e.g., proton and electron transfer, the method has been shown to provide predictive insights into a range of electrocatalytic reactions, including ORR, OER, CO_2_RR, and NRR^100–104^.

It is immediately obvious from the energy diagram in Fig. 6b (blue line) that the ideal catalysts exhibit the same electrochemical potential, equal to the standard reduction potential of the process of interest, for each one-electron transfer step of the overall n-electron transfer process. Any deviation from that balance, leads to an overpotential, as illustrated in Fig. 6b for ORR involving catalysts that bind *OOH too strongly (gray) or too weakly (red)^101,102^. Unfortunately, the energies for one-electron steps cannot be tuned individually because, from the perspective of the surface, adsorbate molecules are very similar and thus bind in similar fashions. For example, any OER/ORR catalyst that increases the energy associated with formation of *OH species will also increase the energy of formation of *OOH intermediates, resulting in scaling relationships with two important consequences. First, the overall catalytic process can be studied using a single descriptor, the Gibb’s free energy of a selected one-electron transfer step. A plot of catalytic overpotential as a function of this descriptor generates well-known volcano plots with the most active catalyst sitting atop the volcano. These binding energies can be further correlated to electronic structure descriptors, which explain bond formation between adsorbate and catalyst. A formative descriptor proposed by Norskov and Hammer is *d*-band theory, which describes adsorbate binding to transition metal catalysts based on position, shape, and filling of the metal *d*-band. Generally, metal *d*-bands which are high in energy, relative to Fermi level (*E*_F_), will generate catalyst-adsorbate antibonding orbitals which are also high in energy, i.e., less filled, making strong interactions and vice versa^105^. The *d*-band theory provides a framework by which we understand catalyst–adsorbate interactions through chemically intuitive electronic structure orbital descriptors. The second important consequence of Norskov’s scaling relationships is that, even for the catalyst at the top of the volcano plot, there may exist an overpotential which cannot be eliminated using simple electrocatalytic transition metal-based surfaces (0.37 V for ORR and OER)^101,102^. This intrinsic overpotential has prompted computational scientists to propose utilization of co-catalysts, that can selectively tune binding energies of individual intermediates^106,107^.

Similar computational approaches have been applied to EN. Activity of transition metals was probed in several nanocarbon metal-free, monometallic, and bimetallic environments, as shown in Fig. 6a. Substrate binding sites for metal-free systems are usually carbon atoms neighboring the heteroatom, metal sites for monometallic species, and one or two metals for bimetallic species (binding sites are marked with arrows, Fig. 6a). Similar scaling relationships were identified, leading to a single descriptor, such as the binding energy for *OH (in OER/ORR) or *N (in NRR) that predicts reactivity and produces volcano plots (Fig. 5c–g). For ORR, the most active investigated catalysts, which reside at the top of the volcano plots are Fe-pyridine-N_4_ (overpotential = 0.42 V, Fig. 6c)^108^, bimetallic CoPtN_8_V_4_ (overpotential = 0.30 V, Fig. 6d)^109^, and N-doped graphene (overpotential = 0.36 V, Fig. 6e)^110^. Additionally, the most active catalysts for the OER and NRR were identified to be Ir-pyrrole-N_4_ SAC (overpotential = 0.30 V, Fig. 6f)^108^ and Ru-g-C_3_N_4_ (overpotential = 0.478 V, Fig. 6g)^111^.

To obtain a better chemical intuition for the observed trends, other descriptors have been identified based on electronic properties of the material. For example, catalytic efficiency for NRR was found to correlate with the integrated crystal orbital Hamilton population (ICOHP) descriptor to model the interaction between an adsorbate atom and a solid surface. Hamiltonian-weighting of the solid density-of-states gives partitioned band structure energies, which are separated into bonding, non-bonding, and anti-bonding states and displayed relative to *E*_F_. Calculating the ICOHP of all states below *E*_F_ provides chemical information about bond strength between the adsorbate and surface. The ICOHP was shown to be well-aligned with ΔG_*N_ and thus activity towards NRR^111^. For a broad range of catalysis, Zeng established a highly convenient, generalized universal descriptor (*φ*) for SACs which showed a linear relationship with, for example, ΔG_*OH_. Briefly, *φ* accounts for the number of *d*-electrons residing on the metal active site after charge redistribution from ligation and intermediate adsorption. *φ* includes contributions of electronegativity (*E*), number of nearest neighbor ligands (*η*) and *d*-electrons (*θ*) from the ligands, adsorbates and metal center. According to the universal descriptor, a larger *φ* results in larger filling of metal *d*-states, which depends on its position relative to *E*_F_^108^.

Chemical selectivity of the catalyst was also investigated, particularly in systems where competing HER is thermodynamically feasible (NRR and CO_2_RR). For example, the key selectivity determining step in NRR vs. HER was identified as binding of *N or *H, where surfaces with strong nitrogen binding are more selective toward NRR. Even though metal surfaces show strong preference for *H binding with few exceptions among early transition metals, SACs exhibit HER suppression because of the ensemble effect^112^. In effect, the ensemble effect, resulting from interactions between single metal atoms and surrounding ligands, precludes H-adsorption to preferred hollow or bridge sites and only allows for top site adsorption. However, HER is still a competing pathway for most SAC^111^. By plotting ΔG_*N_ vs. ΔG_*H,_ one can identify the most selective catalysts for NRR (Fig. 6h), which were found to be Mn-SV-N_3_, V-pyridine-N_4_, and Ti-pyridine-N_4_^112^. Improved selectivity towards NRR of SAC compared to metal catalysts is further rationalized by the difference in mechanisms of HER on those surfaces. On metal catalysts HER proceeds though easier Tafel mechanism with H–H coupling occurring between adjacent metal sites. On SAC that pathway is hindered because adjacent metal centers are far away and do not allow coupling of 2H* species. Therefore, HER follows the more difficult Heyrovsky pathway making NRR relatively competitive with HER.

## Future directions

One of the principal challenges impeding development of superior EN materials is the knowledge gap, which exists between solid heterogenous electrocatalysts and their molecular counterparts. Further challenging the situation are elevated, pyrolytic temperatures, which are often employed for synthesis of graphitic carbons, in which precise control over chemical functionalization is lost making mechanistic investigations implausible. As such, EN with well-characterized chemical structures make ideal platforms for facile mechanistic investigations and should continue to be pursued. Synthetic techniques which circumvent these high temperatures and can produce desired functionalities will make valuable contributions to EN research.

Some inspiring strategies for synthesis of EN worth-exploring use chemical additives that can effectively lower the barrier of graphitic precursor decomposition, and adventitious functional groups on graphitic carbons for surface modifications. For example, when metal fluorides are added during synthesis, they release fluorine and have been shown to improve kinetics associated with carbon feedstock decomposition in the growth of graphene monoliths, revealing a path to reducing extreme temperature parameters.^113^ Surface oxide species, e.g., quinones, carboxylates, and alcohols, native to pre-existing graphitic carbons are common reactive functional groups used in named organic reactions. Making use of these inherent reactive sites, like the reaction between o-quinone groups and 1,2-diamines, are a simple way of covalently grafting molecular sites to conductive carbons. With well-defined catalytic motifs, fundamental studies of heterogenous ISET have been made possible^114^.

Further enhancement of spectroscopic and microscopic methods, both in situ and ex situ, for probing EN before, during and after catalysis will be of benefit to our mechanistic knowledge. Undoubtedly, coupling together complementary techniques, i.e., hyphenated techniques, may radically improve structural characterization of EN. For example, synchrotron X-ray scanning tunneling microscopy (SX-STM) combines together the elemental sensitivity of X-rays and sub-nanometer spatial resolution of STM^115^. Spectroscopic signatures belonging to adsorbed intermediates can be detected via, for example, Raman and FTIR spectroscopies, however, the observed state will be that of the kinetically slowest intermediate. The main bottleneck to improved spectroelectrochemical investigations is the limitations on temporal resolution of the chosen spectroscopy—chemical processes which are being monitored are happening at a much faster timescale than what can feasibly be sampled. If time-resolutions of optical instruments can continue to be improved, the range of catalytic intermediates we are able to observe may grow^116^.

## Data Availability

Data sharing not applicable as no datasets were generated or analyzed during the current study.
